# The Levels of Ghrelin, TNF-α, and IL-6 in Children with Cyanotic and Acyanotic Congenital Heart Disease

**DOI:** 10.1155/2007/32403

**Published:** 2007-09-04

**Authors:** Erdal Yilmaz, Bilal Ustundag, Yasar Sen, Saadet Akarsu, A. Nese citak Kurt, Yasar Dogan

**Affiliations:** ^1^Division of Pediatric Cardiology, Department of Pediatrics, School of Medicine, Firat University, Elazığ 23119, Turkey; ^2^Department of Biochemistry, School of Medicine, Firat University, Elazığ 23119, Turkey; ^3^Division of Pediatric Endocrinology, Department of Pediatrics, School of Medicine, Firat University, Elazığ 23119, Turkey; ^4^Department of Pediatrics, School of Medicine, Firat University, Elazığ 23119, Turkey

## Abstract

*Background/Aim*. Ghrelin has effects on nutrient intake and growth. The cause of growth retardation in congenital heart disease is multifactorial. The aim of the present study is to investigate the ghrelin in congenital heart disease and the association of ghrelin with TNF-α and IL-6. *Materials and methods*. We measured serum ghrelin, TNF-α, and IL-6 levels using spesific immunoassay in 68 patients (47 acyanotic, 21 cyanotic with congenital heart disease) and in 25 control subjects. *Results*. In comparison to controls, serum ghrelin, TNF-α levels were significantly higher in acyanotic patients and cyanotic patients with congenital heart disease (P<.0001). In acyanotic and cyanotic patients with congenital heart disease, there was a positive correlation between ghrelin and TNF-α (r=.485, P<.05 and r=.573, P<.01, resp.). *Conclusion*. Serum ghrelin levels is elevated in acyanotic and cyanotic patients with congenital heart disease. Increased ghrelin levels represents malnutrition and growth retardation in these patients. The relation of ghrelin with cytokines may be explained by the possible effect of chronic congestive heart failure and chronic shunt hypoxemia.

## 1. INTRODUCTION

Ghrelin, a 28-amino-acide peptide, is a potent stimulator of growth hormone release that has been implicated in the control of food intake and energy homeostasis in human begins and rodents [1–5]. Ghrelin is mainly produced in the stomach. Ghrelin is not secreted into the gastrointestinal tract like digestive enzymes but into blood vessels to circulate throughout the body [6]. Ghrelin causes weight gain by increasing food intake and reducing fat use [7, 8]. Ghrelin has effects on nutrient intake and growth hormone (GH) release, subsequently on physical development and growth [9].

Tumor necrosis factor α (TNF-α) and interleukin-6 (IL-6) are pleiotropic cytokines with numerous immunologic and metabolic actions [10, 11]. IL-6 is generally considered to be an important cytokine in the network of cytokines that regulate immune reactions and acute phase responses [12].

The relationship between congenital heart disease (CHD), malnutrition, and growth retardation is well documented [13]. Infants with congenital heart disease are prone to malnutrition for several reasons including decreased energy intake, increased energy requirements, or both. Different types of cardiac malformations can affect nutrition and growth to varying degrees [14]. Although nutritional and growth status were investigated in children with cyanotic and acyanotic heart disease, serum ghrelin levels have not been established. The objective of this study is to investigate and compare the functional role of ghrelin on the regulation of energy balance in children with cyanotic and acyanotic congenital heart disease and the association of ghrelin with TNF-α, IL-6, that were not entirely confirmed in literature by now.

## 2. MATERIAL AND METHODS

### 2.1. Study population

The study was conducted on 47 children with acyanotic CHD, 21 children with cyanotic CHD, and 25 healthy children. All patients' cardiac diagnoses were made on the basis of clinical and laboratory examinations. None of the patients had associated abnormalities or pulmonary hypertension. Body mass index (BMI) was calculated as the ratio of body weight (kg) and squared height (m). The local ethics committee approved the study protocol. Informed consents were obtained from the parents of the subjects.

### 2.2. Laboratory investigation and immunoassay

All blood samples were drawn at 08-09 am and stored −20°C until the procedure. Serum ghrelin, TNF-α, and IL-6 levels were analyzed with ELISA kits (TNF-α, IL-6 kit was purchased from Bio-Source International Inc. (Camarillo, Calif, USA); Ghrelin kit from Phoenix International, Inc, USA).

### 2.3. Statistical analyses

All data were analyzed by SPSS software, version 10.0 for Windows. Data were presented as mean ± standard deviation. The given data were compared between groups using one-way ANOVA, followed by Post-hoc; Bonferroni test. Correlation between the parameters were explored with Spearman's correlation. P values less than .05 were considered statistically significant.

## 3. RESULTS

In 47 acyanotic patients, mean age was 30.5±18.4 months, in 21 cyanotic patients was 28.4±15.6 months and in 25 control subjects was 31.1±15.1 months. Age and anthropometric data of the patients and the control subjects are shown in Table 1. There was no significant difference between groups (the acyanotic patients, the cyanotic patients) in terms of mean age, weight, height, BMI. The specific cardiac lesions of patients are listed in Table 2.

Serum ghrelin levels were significantly higher than in acyanotic and cyanotic groups compared to in the control group (P<.0001) (Table 3). Serum ghrelin levels in the acyanotic patients were significantly higher than in the cyanotic patients (P<.0001). TNF-α levels were significantly higher than in cyanotic and acyanotic patients with CHD compared to in the control groups (P<.001, P<.0001, resp.). Serum TNF-α values were higher in the acyanotic patients compared to the cyanotic patients with CHD (P<.001). Serum IL-6 levels were higher than in cyanotic and acyanotic patients with CHD compared to in the control groups (P<.0001, P<.001, resp.).

**Figure 1:**
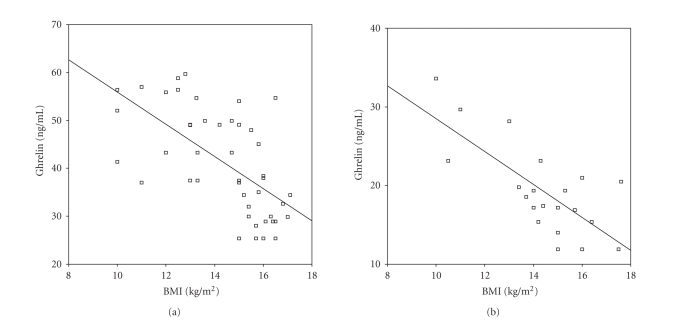
(a) Correlation of ghrelin with BMI in acyanotic patients with CHD (r=−.549, P<.05). (b) Correlation of ghrelin with BMI in cyanotic patients with CHD (r=−.688, P<.01).

**Figure 2:**
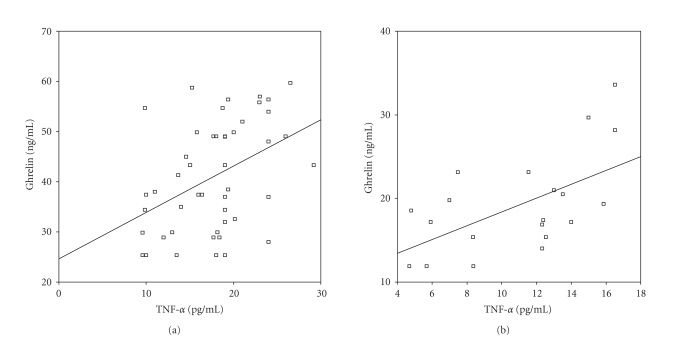
(a) Correlation of ghrelin with TNF-α in acyanotic patients with CHD (r=.485, P<.05). (b) Correlation of ghrelin with TNF-α in cyanotic patients with CHD (r=.573, P<.01).

In both acyanotic and cyanotic groups, serum ghrelin levels were negatively correlated with BMI (r=−.549, P<.05 and r=−.688, P<.01, resp.) (Figures 1(a) and 1(b)). IL-6 and TNF-α levels were not related to BMI in the acyanotic and cyanotic patients with CHD. Ghrelin levels were also correlated with TNF-α in the acyanotic and cyanotic groups (r=.485, P<.05 and r=.573, P<.01, resp.) (Figures 2(a) and 2(b)). Ghrelin levels were not related to IL-6 in the acyanotic and cyanotic patients with CHD (r=−.263, P>.05 and r=.398, P>.05, resp.).

## 4. DISCUSSION

The cause of growth retardation in CHD is multifactorial. Inadequate caloric intake, malabsorption, and increased energy requirements caused by increased metabolism may all contribute. However, inadequate caloric intake appears to be the most important cause of growth failure in CHD [13, 15, 16]. Patients with acyanotic heart disease had a greater growth deficit in weight, and those with cyanotic heart disease had a greater growth deficit in stature as demonstrated by both decreased height and weight. Although growth impairment is most pronounced in infants with cyanotic CHD, growth failure does not correlate well with the degree of hypoxia. In this study, the cyanotic patients had a more pronounced retardation in both height and weight than in the acyanotic patients [13, 17].

Ghrelin is accepted as a good marker of the nutritional state, mainly in situations of malnutrition, like anorexia nervosa, owing its fast recovery after weight gain [18]. The inverse correlation between ghrelin levels and BMI is well defined [9, 19]. We observed the mentioned correlation, both in children with cyanotic heart disease and in children with acyanotic heart disease.

Although the cyanotic patients had a more pronounced retardation in both height and weight than in the acyanotic patients, we found that serum ghrelin levels significantly elevated in the acyanotic patients than in the cyanotic patients (P<.0001). Growth failure in cyanotic children has not been shown to be proportional to the severity of cyanosis, suggesting that multiple factors are involved in the pathogenesis of their growth disturbance [20]. Alteration of endocrine mediators of growth has been implicated as a possible mechanism of growth failure in cyanotic patients. Cyanotic newborn lambs have decreased levels of serum insulin-like growth factor I without a corresponding decrease in growth hormone or hepatic growth factor receptors [21]. Weintraub et al. [22] reported that while insulin-like growth factor I levels were linearly related to height and weight in patients with cyanotic lesions, no such correlation was found in their cyanotic patients. These studies suggest that chronic tissue hypoxia may have independent role in growth failure.

We found that serum TNF-α significantly increased in the cyanotic patients and in the acyanotic patients. Similarly, serum IL-6 was increased in both groups but the change was more distinctive in the cyanotic patients. TNF-α and IL-6 appear to be important cachectic process mediators, although this association is not completely established [23, 24]. Cardiac cachexia describes wasting primarily due to loss of lean body mass. Cachexia results in decreased muscle strength and function and compromised immune function [25, 26]. This syndrome is likely to occur in children who have chronic congestive heart failure, chronic shunt hypoxemia [27]. In addition to inadequate calorie and protein intake, there is evidence that this syndrome may be caused by circulating tumor necrosis factor, which stimulates catabolism [28].

In the present study, ghrelin correlated to positively with TNF-α, in acyanotic patients and cyanotic patients with CHD. The relation of ghrelin with TNF-α raises the possibility of the direct effect of TNF-α upon ghrelin or the impact of heart failure severity upon both ghrelin and TNF-α. Nagaya et al. [29] have shown that plasma ghrelin level is increased in cachectic patients with congestive heart failure as a compensatory mechanism in response to anabolic-catabolic imbalance.

In conclusion, serum ghrelin level is elevated in cyanotic and acyanotic patients with CHD. Increased ghrelin levels represents malnutrition and growth retardation in these patients. Additionally, the relation of ghrelin with cytokines may be explained by the possible effect of chronic congestive heart failure and chronic shunt hypoxemia.

## Figures and Tables

**Table 1:** Age and anthropometric data of the patients and the control subjects.

	Cyanotic patients	Acyanotic patients	Control
	(n=21)	(n=47)	(n=25)
Age (month)	30.5±18.4	28.4±15.6	31.1±15.1
Female/Male ratio	10/11	30/17	15/10
Weight (kg)	9.7±7.4	10.9±6.2	21.2±9.9
Height (cm)	76.8±26.8	83.6±21.9	98.4±22.9
BMI (kg/m^2^)	14.9±2	14.6±2.1	21.5±5.5

**Table 2:** Diagnosis of the patients.

Diagnosis	No.
***Cyanotic patients***	
Tetralogy of Fallot	15
Tricuspid atresia	3
Transposition of great arteries	2
Truncus arteriosus	1
***Acyanotic patients***	
Ventricular septal defect	35
Atrial septal defect	11
Patent ductus arteriosus	1

**Table 3:** Ghrelin, TNF-α, and IL-6 levels of patients with CHD and control groups.

	Cyanotic patients	Acyanotic patients	Control
	(n=21)	(n=47)	(n=25)
Ghrelin (ng/ml)	$19.2\pm 5.9^{\text{b},\text{c}}$	$41.9\pm 11.6^{\text{a}}$	8.6±1.7
TNF-α (pg/ml)	$11\pm 4.1^{\text{e},\text{f}}$	$18.8\pm 5.4^{\text{a}}$	7.5±3.4
IL-6 (pg/ml)	$16.2\pm 6.9^{\text{b}}$	$13.6\pm 5.8^{\text{d}}$	3.2±1.4

${}^{\text{a}}P<.0001$ control: acyanotic group

${}^{\text{b}}P<.0001$ control: cyanotic group

${}^{\text{c}}P<.0001$ cyanotic: acyanotic group

${}^{\text{d}}P<.001$ control: acyanotic group

${}^{\text{e}}P<.001$ cyanotic: acyanotic group

${}^{\text{f}}P<.01$ control: cyanotic group
